# Effect of anti-obesity agent HSG4112 on overweight and obese patients following 12 weeks of oral treatment: a study protocol for a randomised, double-blind, placebo-controlled, parallel-group, phase 2a clinical trial

**DOI:** 10.3389/fphar.2023.1177539

**Published:** 2023-08-24

**Authors:** Kyungha Min, Bumjo Oh, Hye Yeon Koo, Yang-Hyun Kim, Ji-Won Lee, Sangsub Lee, Youngah Kim, Hyuktae Kwon

**Affiliations:** ^1^ Department of Family Medicine, Seoul National University College of Medicine and Seoul National University Hospital, Seoul, Republic of Korea; ^2^ Department of Family Medicine, Seoul Metropolitan Government-Seoul National University Boramae Medical Centre, Seoul, Republic of Korea; ^3^ Department of Family Medicine, Seoul National University Bundang Hospital, Seongnam, Republic of Korea; ^4^ Department of Family Medicine, Korea University College of Medicine, Seoul, Republic of Korea; ^5^ Department of Family Medicine, Severance Hospital, Yonsei University College of Medicine, Seoul, Republic of Korea; ^6^ Institute for Innovation in Digital Healthcare, Yonsei University, Seoul, Republic of Korea; ^7^ Glaceum Incorporated, Suwon, Republic of Korea

**Keywords:** obesity, overweight, weight loss, obesity drug, randomised controlled trial

## Abstract

Background: Glaceum Inc. has proposed HSG4112, a structural analogue of glabridin, as a novel anti-obesity compound. Animal studies and phase I human trials have shown that HSG4112 improves energy consumption, normalises weight, and is safe and drug-resistant. Based on these results, the company plans to conduct a phase 2a clinical trial to determine the safety and efficacy of HSG4112 in overweight and obese patients. Methods: A 16-week randomised, double-blind, placebo-controlled, parallel-group trial will be conducted at five large hospitals in South Korea to assess the safety and efficacy of HSG4112 in overweight and obese patients. Participants who meet the inclusion/exclusion criteria will be assigned a subject number and randomly assigned to one of the four treatment groups (one group receiving a placebo) in a 1:1:1:1 ratio. The study’s primary outcome will be to monitor the change in body weight (kg) from baseline to the end of treatment while monitoring safety and tolerability. Discussion: This trial will evaluate the efficacy and safety of HSG4112 in overweight and obese adults. Upon proving the safety and effectiveness of the newly developed mechanism, it might significantly improve the perception of the product among medical personnel and obese patients. Furthermore, it may aid in managing chronic conditions that require long-term treatment.

**Trial registration:** ClinicalTrials.gov, identifier [NCT05197556].

## 1 Introduction

Obesity is defined as abnormal or excessive fat accumulation that can adversely affect health (Panuganti et al., 2022). World Health Organization has reported that overweight and obesity increase the risk of non-communicable diseases. In the past 45 years, the rate of increase in obesity has tripled worldwide, with 1.9 billion adults aged 18 years or above being overweight and 650 million classified as obese (Ghaus et al., 2021). Obesity is associated with a high risk of morbidity, disability, and death, with mortality risk similar to that of smoking (Whitlock et al., 2009). Major risk factors for mortality include an increased incidence of cardiovascular events, such as coronary heart disease, hypertension, stroke, type 2 diabetes, and some types of cancer, including breast cancer in postmenopausal women, endometrial cancer, and colon and kidney cancer (Knight, 2011). The risk of these non-communicable diseases increases with increased body mass index (BMI). Moreover, reports show that obesity is positively correlated with an increased risk of morbidity and mortality related to COVID-19 (Singh et al., 2022).

Several studies have confirmed that lifestyle changes, particularly reducing body weight and BMI through changes in eating habits and increasing physical activity, can lower the risk factors associated with obesity-related diseases, such as coronary heart disease (Całyniuk et al., 2016). Accordingly, the weight loss industry, recognizing obesity as a disease, is continuously growing. Various methods have been used to lose weight and reduce the risk of chronic diseases. Due to fewer side effects, behavioural and dietary modifications and increased exercise are considered the first treatment options for weight loss in obese patients. Drug therapy is recommended for patients who do not respond to lifestyle interventions alone, especially if bariatric surgery is not an option (Jackson et al., 2015). However, oral anti-obesity drugs are currently limited due to concerns about their efficacy and drug resistance (Tak and Lee, 2021).

Clinicians urgently require drugs that facilitate efficient weight loss. HSG4112, a structural analogue of glabridin, has a novel anti-obesity mechanism. The results of the anti-obesity trial, which includes HSG4112 as a primary indicator for anti-obesity treatment, along with the anti-inflammatory trial of HSG4112, confirmed that HSG4112 not only effectively reduces body weight but also improves inflammation, thereby promoting fat metabolism as an anti-obesity agent. Furthermore, it has been determined through safety and pharmacological testing that HSG4112 does not impact the central nervous, respiratory, and cardiovascular systems (Choi et al., 2021).

Several phase I trials were conducted to confirm that HSG4112 was safe and drug-resistant in humans. The results are as follows:

In the HSG4112-P1-01 trial, healthy male subjects were enrolled to evaluate the safety, tolerability, pharmacokinetic/pharmacodynamic characteristics, and food effect on HSG4112 following single and repeated oral administration. Safety and tolerance in all dosage groups were satisfactory.

In the HSG4112-P1-02 trial, healthy adult male subjects were enrolled to investigate the impact of food on the pharmacokinetic characteristics of HSG4112. The study involved a single-dose administration of HSG4112 and utilised a 3-way crossover design. Safety evaluation showed no clinically significant safety or tolerability-related issues; it was determined that the presence or absence of diet (fasting or fed condition) and type of diet (low-fat or high-fat diet) did not affect safety or tolerability.

The HSG4112-P1-03 study is ongoing to evaluate the safety of HSG4112 oral administration in 480 mg and 720 mg dosages in healthy and obese adult subjects, including female subjects. In the Part 1 study, the safety and tolerance in healthy adult female subjects were satisfactory. The Part 2 study is also ongoing in obese adult subjects.

## 2 Methods/design

### 2.1 Study design

**FIGURE 1:**
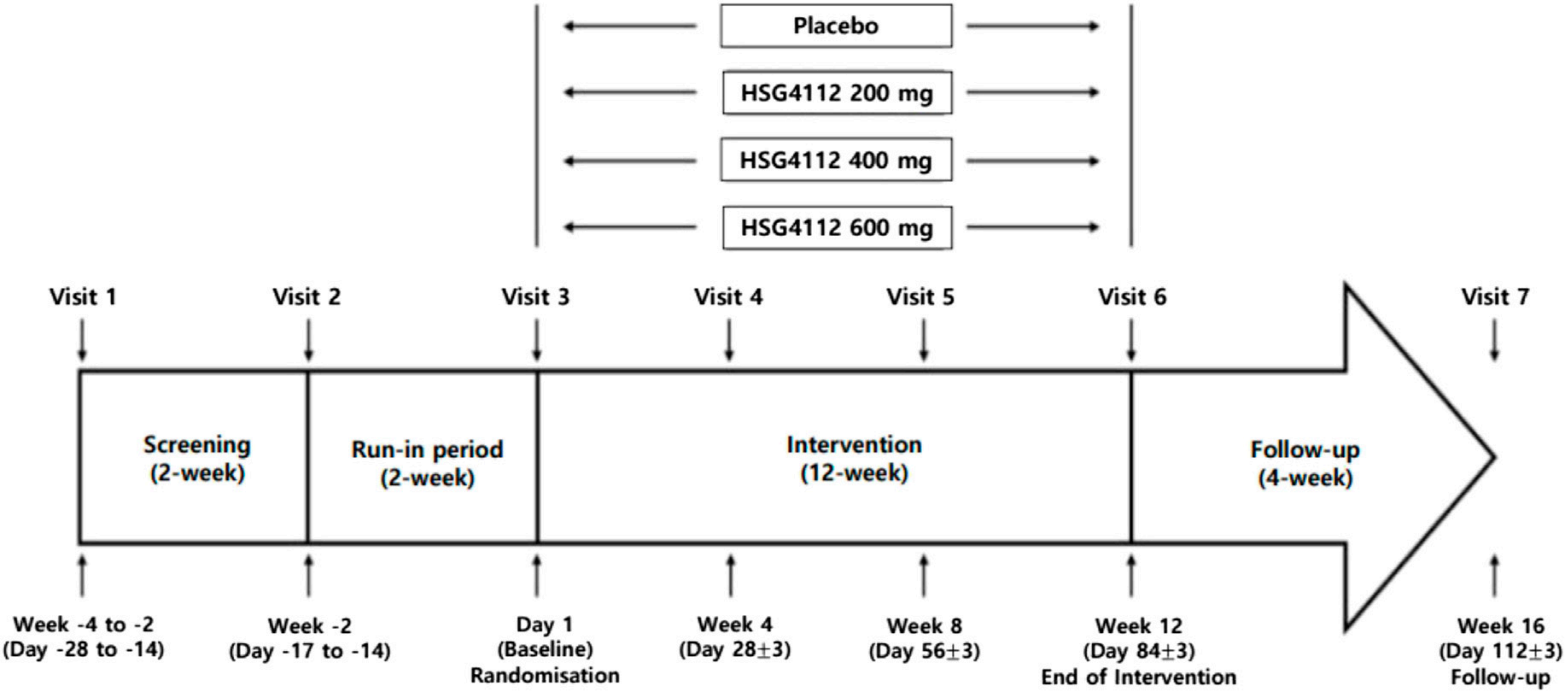
Flow diagram of the trial.

This will be a randomised, double-blind, placebo-controlled, parallel-group trial. This study will be conducted over 16 weeks (intervention for 12 weeks) at five large hospitals in South Korea. Participants who are deemed eligible to participate in this study based on the inclusion/exclusion criteria will be assigned a subject number and randomly assigned to one of the four treatment groups. The experimental group was divided into 3 groups in proportion to the dose of HSG4112 compared to the placebo group, and a total of 4 groups were assigned with equal numbers to each group. Approximately 80 eligible participants will be recruited and divided into four groups. The flow diagram of the trial is shown in Figure 1.

### 2.2 Study population—sample size calculation

This study describes a phase 2a clinical trial to evaluate safety and efficacy after repeated administration of HSG4112. Subject sample size estimates for the primary efficacy endpoint are based on total body weight, assuming repeated measurements of body weight at baseline and every 4 weeks after that during the 12 weeks. Based on similar weight loss clinical trials with anti-obesity drugs, the covariance matrix of weight change over time is estimated to be AR(1) (sigma = 3.07, rho = 0.9). Therefore, if 20 subjects complete each administration group in this study, an average difference of 6 kg between HSG4112 and placebo administration can be derived with 80% power, assuming a two-sided significance level of 0.05. A difference of 6 kg is expected to be sufficient to assess the potential efficacy of HSG4112 treatment in this study. Therefore, this clinical trial’s target number of subjects per group was 20 (Muller and Curtis, 1989; Muller et al., 1992; Naik and Rao, 2001; Muller et al., 2007; Simpson et al., 2010).

### 2.3 Study population—inclusion/exclusion criteria

The study population will be adults aged between 19 and 70 years. We will recruit adults with a BMI between 30 and 39.9 kg/m^2^ (obese) with or without comorbid conditions or a BMI between 27 and 29.9 kg/m^2^ (overweight) with at least one documented treated or untreated comorbid condition (e.g., hypertension, dyslipidaemia, cardiovascular disease, glucose intolerance, and sleep apnoea). Other detailed inclusion and exclusion criteria are described in the Supplement.

### 2.4 Recruitment and screening

Participants will be recruited using advertisements and posters in five centres, nearby areas, and online. This study will be described briefly in advertisements and posters, along with the investigator’s contact information.

Potential participants will receive detailed information about the study and have informed discussions with a trained research coordinator. After signing an informed consent form, those who wish to participate are assessed. The screening test will include the participant’s height, weight, vital signs, 12-lead ECG, and laboratory tests. Participants who meet the eligibility criteria will begin a 2-week single-blind run-in period after the screening period. During this period, participants must consume a placebo to determine whether they comply with diet and lifestyle guidelines.

### 2.5 Randomisation, allocation concealment, and blinding

Upon completion of the run-in period, participants will be randomly assigned to one of four treatment groups—one group receiving placebo—in a ratio of 1:1:1:1. The randomisation procedure will be stratified using BMI by the order as follows:

- Randomisation No.: RX### (e.g., R1001)
  - X: BMI (<30 kg/m2 - 1, ≥30 kg/m2 - 2)
  - ###: Placement order of subject participating in this study

Allocation concealment will be maintained because no sealed envelopes will be opened during the study. Group assignments will not be disclosed or made available to participants and investigators throughout the study.

### 2.6 Intervention

In accordance with the study protocol, participants will be randomly assigned to double-blind treatment groups and receive a once-daily oral dose of the investigational product (IP) for 12 weeks. Participants in the four treatment groups will take three tablets daily as follows:

• 1st group: HSG4112 200 mg (1 tablet of HSG4112 and 2 tablets of placebo)
• 2nd group: HSG4112 400 mg (2 tablets of HSG4112 and 1 tablet of placebo)
• 3rd group: HSG4112 600 mg (3 tablets of HSG4112)
• 4th group: placebo (3 tablets of placebo)
* Each HSG4112 tablet includes 200 mg of HSG4112.

The tablets will be dispensed and retrieved by trained research coordinators. The principal investigator and research coordinator will be responsible for storing and maintaining records of the test and placebo products.

### 2.7 Efficacy and safety assessment

The body weight and obesity/metabolism-related parameters will be evaluated to assess the efficacy of HSG4112. Assessments including measurement of vital signs, 12-lead ECG, clinical laboratory tests, pregnancy tests, physical examination, and adverse event monitoring will be performed to evaluate the safety and tolerability of HSG4112. Blood samples will be collected for pharmacokinetic assessment. In addition, samples from participants who have signed consent forms for exploratory genetic research will be analysed to detect polymorphisms in the PON2 gene. A summary of the schedule and all measurements that would be performed, is presented in Table 1.

**TABLE 1:** Schedule of data collection (* Annotations 1–19: Detailed information is described in the Supplementary Data sheet S1).

Assessed items	Screening	Run-in	Intervention			End of Intervention^1^	Follow-up^1^
	Week −4 to −2 (Day −28 to −14)	Week −2 (Day −17 to −14)	Day 1 (Baseline)	Week 4 (Day 28 ± 3)	Week 8 (Day 56 ± 3)	Week 12 (Day 84 ± 3)	Week 16 (Day 112 ± 3)
Visit	1st^2^	2nd	3rd	4th	5th	6th	7th
Informed consent	O						
Eligibility assessment	O		O				
Demographic information	O						
Randomization^3^			O				
Dispensation of IP/placebo^4^		O	O	O	O		
Restitution of IP/placebo^4^			O	O	O	O	
Distribution of Diet/Physical activity diary^5^		O	O	O	O		
DEXA^6^			O			O	
Body weight	O	O	O	O	O	O	O
BMI^7^	O		O	O	O	O	O
Questionnaire (IWQOL-Lite-CT)			O			O	
Anthropometrics^8^	O		O		O	O	
Adverse events and Concomitant drug	O	O	O	O	O	O	O
Physical examination^9^	O		O	O	O	O	O
Vital sign^10^	O	O	O	O	O	O	O
12-lead ECG	O		O		O	O	
Clinical laboratory tests^11^	O		O	O	O	O	
Drug abuse test^12^	O						
Serum TSH	O						
Serologic test^13^	O						
Pregnancy test^14^	O		O	O	O	O	
Sex hormone test^15^	O		O		O	O	
Genotyping sampling^16^			O				
Pharmacokinetic sampling^17^			O	O	O	O	
Pharmacodynamic sampling^18^			O		O	O	
Body composition measurement^19^			O	O	O	O	

The primary outcome will be as follows:

• Change in Body Weight (kg) from Baseline to End of Treatment.
• Evaluation of safety and tolerability by monitoring adverse events (number of adverse events, adverse drug reactions, serious adverse events, and suspected unexpected serious adverse reactions).

The secondary outcome will be as follows:

• Percentage of Participants with ≥5% Body Weight Loss from Baseline to End of Treatment.
• Percentage of Participants with ≥7.5% Body Weight Loss from Baseline to End of Treatment.
• Percentage of Participants with ≥10% Body Weight Loss from Baseline to End of Treatment.
• Percentage change in body weight from baseline to the end of treatment.

Additional exploratory analysis and safety analysis will be conducted.

### 2.8 Statistical analysis

The statistical analyses will be conducted by well-trained statisticians independent of the research team. Data will be analysed using intention-to-treat or per-protocol analyses. To compare the week 12 weight change from the baseline, each group will be analysed using the mixed model of repeated measures. A sensitivity analysis will be conducted using covariance analysis and model analysis. To compare the percentage change in body weight from baseline to the end of treatment, a logistic regression model will be used. Statistical *p*-value < 0.05 will be considered statistically significant. All analyses will be performed using the SAS software.

### 2.9 Data collection and quality control

The data for this study will be collected using electronic case-report forms. A computerised case report system will be used to enter data following each hospital visit. Furthermore, periodic monitoring will be carried out to ensure high accuracy and quality throughout the study.

### 2.10 Handling of withdrawal

During the study, participants will be free to withdraw their consent for any reason at any time. If the participant wishes to withdraw from the study, the investigator will request a reason for the withdrawal. Dropout participants will complete follow-up visits for at least 4 weeks after taking the last test drug. However, concomitant drugs and adverse events will be checked via telephone if the participant refuses to visit.

## 3 Discussion

Appetite-suppressing drugs currently dominate the obesity treatment market. The recently developed drugs emphasise the role of GLP-1 in diabetes treatment and have proven effective. However, they do have limitations in terms of safety, mainly posing risks associated with cardiovascular side effects.

If the newly developed mechanism is safe and effective, a significant reduction is anticipated regarding the limitations in the perception of medical personnel (suppliers) and obese patients (consumers) when choosing to take oral drugs, along with the transition to chronic conditions that require long-term management.

Prior to this, the significant weight loss and safety of the treatment can be confirmed by comparing it with a placebo control group, which has been confirmed to be effective in animal and phase one human trials.

## 4 Trial status

The recruitment phase of the trial began in February 2022. As per the protocol, 81 participants were divided into four groups. We anticipate that data collection and analysis will be completed by 2023. Protocol modifications will be communicated to all relevant parties, and the Clinical Trials Registry will be updated accordingly (ClinicalTrials.gov).

## Data Availability

The original contributions presented in the study are included in the article/Supplementary Material, further inquiries can be directed to the corresponding author.
