# Supplementary material for: Effect of anti-obesity agent HSG4112 on overweight and obese patients following 12 weeks of oral treatment: a study protocol for a randomised, double-blind, placebo-controlled, parallel-group, phase 2a clinical trial
Source: Front Pharmacol. 2023 Aug 24;14:1177539. doi: 10.3389/fphar.2023.1177539 (PMC10483829; doi:10.3389/fphar.2023.1177539)
Supplement: Supplementary file 1 [file DataSheet1.pdf]

Supplements

Study population - Inclusion Criteria

1) Prior to the screening, participants must be able to comprehend and sign an informed consent form approved by the IRB.

2) At the time of screening, the participant must be between 19 and 70 years, inclusive.

3) The BMI should be between 30 and 39.9 kg/m^2, inclusive (obese), with or without comorbid conditions. The BMI should be between 27 and 29.9 kg/m^2, inclusive (overweight), with at least one documented treated or untreated comorbid condition (e.g., hypertension, dyslipidaemia, cardiovascular disease, glucose intolerance, and sleep apnoea). All comorbid conditions were considered clinically stable by the investigator.

   - (BMI (kg/m^2) = weight (kg) / {height $(m)^2$})

4) The following are the requirements for females:

   - Females of childbearing potential who are not pregnant, evidenced by a negative serum hCG pregnancy test at screening.

   - Non-lactating, surgically sterile (defined as documented bilateral tubal ligation, bilateral tubal occlusion, bilateral oophorectomy, hysterectomy), or postmenopausal (defined as at least 12 months prior to screening without menses without an alternative explanation for the absence of menses).

5) Eligible males must not have planned to have children within 90 days after the last day of treatment.

6) At least one unsuccessful weight loss attempt, as determined by the investigator.

Study population - Exclusion Criteria

1) Clinically significant newly diagnosed illness, per investigator judgment, 1 month before screening and during the screening period.

2) Significant history or clinical evidence of allergic reactions or hypersensitivity to IP or any related drug compound.

3) Compliance with placebo self-administration was ≤80% during the initial 2-week single-blind placebo run-in period.

4) Weight loss >3% during the 2-week single-blind placebo run-in period.

5) Diabetes mellitus (type 1, type 2, or others). A remote history of gestational diabetes that had been resolved was not excluded.

6) Previous or planned (during the study) bariatric surgery or device (i.e., gastric bypass, gastric banding, sleeve gastrectomy, gastric balloon, biliopancreatic diversion)

7) An anticipated surgical procedure during the study period may interfere with the completion of the study or compliance with the protocol.

8) Uncontrolled hypertension at screening was defined as systolic blood pressure ≥ 160 mmHg or diastolic blood pressure ≥ 100 mmHg. Participants with uncontrolled hypertension at screening may undergo re-screening no later than 3 months after initiating or adjusting antihypertensive therapy if enrolment has not been closed.

9) Any of the following within 3 months of screening: myocardial infarction, unstable angina, cerebrovascular accident, transient ischaemic attack, or cardiac arrhythmia that requires medical or surgical treatment.

10) Evidence of any other unstable or untreated clinically significant hepatic, renal, neurological, immunological, respiratory, endocrine, haematological, cardiovascular, psychiatric, or neoplastic diseases or conditions that, in the investigator's opinion, would render the participant ineligible for the study.

11) Males with the following conditions:

- History of or known cause of hypogonadism (e.g., treatment for prostate cancer)
- History of infertility
- Klinefelter or Kallmann syndrome
- Any medication taken within 6 months of screening that can alter reproductive hormone levels, either as an intended effect or as a side effect. These include anabolic steroids, androstenedione, bicalutamide, cimetidine, dehydroepiandrosterone, diethylstilbestrol, other oestrogens, dutasteride, finasteride, glucocorticoids (e.g., prednisone, cortisone, hydrocortisone, and decadron), oral ketoconazole, megestrol acetate, opiates (e.g., morphine, codeine, oxycodone, and hydrocodone), spironolactone, testosterone, or any androgen, and any medications for treating prostate cancer.

12) Major surgical procedures (intrathoracic, intracranial, intraperitoneal, liposuction) within 6 months of the screening visit.

13) New York Heart Association class III and IV congestive heart failure.

14) Organ transplantation history.

15) Screening for TSH greater than two times the upper limit of normal. Participanits on L-thyroxine replacement were required to be on a stable dose for at least 6 weeks before screening.

16) Hyperthyroidism (screening laboratory value TSH < lower limit of normal) and/or participants taking methimazole, carbimazole, propylthiouracil, and/or beta-blockers for hyperthyroidism.

17) Fasting triglycerides > 500 mg/dL at screening. If enrolment is not closed, participants with elevated triglyceride levels at screening may be re-screened after 3 months of initiating or adjusting lipid-lowering treatment.

18) Screening glycosylated haemoglobin (HbA1c) ≥ 6.5%.

19) Fasting plasma glucose ≥ 126 mg/dL.

20) The presence of clinically significant abnormalities in hepatic (e.g., AST or ALT exceeding 2.5x ULN, or total bilirubin levels exceeding 2x ULN, unless Gilbert's syndrome has been documented) or renal function laboratory tests (e.g., glomerular filtration rate < 60 mL/min).

21) A positive HBsAg, HCV Ab, HIV Ag/Ab, or VDRL result at screening.

22) Malignancy within five years of the screening visit (except for adequately treated cutaneous basal cell or squamous cell carcinoma).

23) A stable dose (and dosing interval) of all chronic medications (including, but not limited to, antihypertensives, lipid-lowering drugs, cardiovascular drugs, and antidepressants) must be taken at least 3 months prior to screening.

24) Treatment within 1 month of the screening visit with over-the-counter weight loss products or appetite suppressants (including herbal weight loss agents), St. John's Wort, or within 3 months with an approved anti-obesity drug.

25) Recent (within 6 months of screening) participation in any organised weight loss program.

26) Treatment with systemic (i.e., oral or intravenous) steroids for 7 days within 3 months of the screening visit.

27) Recent history (i.e., within two years prior to the screening visit) of alcohol or drug abuse or a positive drug test at screening. Participants with a positive drug screen may be eligible with approval from the sponsor. This is if the participant has a documented medical history requiring chronic pain treatment and a documented concomitant medication resulting in a positive drug screen. Additionally, the investigator considers the participant to be reliable for this study.

28) Significant changes in smoking habits within 3 months prior to screening, as determined by the investigator.

29) The participant has participated in any other clinical or bioequivalence study and has been dosed with an IP within 6 months before the first day of treatment. Unless documentation indicates that the participant received a placebo, the participant is ineligible.

30) Significant changes in diet or level of physical activity within 1 month prior to screening, based on the investigator’s judgment.

31) Weight loss or gain of more than 5% within 3 months prior to the screening visit by history or documentation.

32) The participant is unwilling, or whose partner is unwilling, to use medically acceptable contraception during and for 90 days following completion or withdrawal of the study.

Medically acceptable contraceptive methods include the following:

- Hormonal contraceptive
- Intrauterine device that has been demonstrated to be effectively used by the participant or the participant's spouse/partner.
- Physical contraception (male or female) used with chemical sterilisation.
- Surgical sterilisation of the participant or the participant's partner (e.g., vasectomy, hysterectomy, tubal ligation, salpingectomy).

33) Participants who, in the opinion of the investigator, should not participate in this study based on clinical laboratory test results or other reasons.

Schedule of data collection – Detailed description

1) In cases of early termination of follow-up or discontinuation of the study, the follow-up visit was performed 4 weeks after the last IP administration.

2) The first and second procedures can be performed on the same day. However, the tests

and procedures to be performed at the screening visit must be completed prior to the suitability evaluation and randomisation of the inclusion/exclusion criteria.

3) Randomisation: Eligible participants were stratified according to baseline BMI and randomised in a 1:1:1:1 ratio into four treatment groups on day 1.

4) Dispensation and restitution of IP/placebo: Participant compliance was evaluated based on the quantity of test drugs delivered to the participants and the number of test drugs returned.

5) Distribution Diet/Physical Activity Diaries: Between visits two and five, diaries were distributed to participants to record their food intake and physical activity per the instructions.

6) DEXA: The result on day 1 may be replaced if there is a measurement result between days 1 and 17.

7) BMI calculation: Height is determined during the first visit.

8) Anthropometrics: Measure the circumference of the waist and the waist-to-hip ratio.

9) Physical examination: Includes evaluation of the skin, head, eyes, ears, nose, throat, neck, thyroid, lungs, cardiovascular system, abdomen, lymph nodes, musculoskeletal system, and extremities.

10) Vital signs were measured, including temperature, blood pressure, and heart rate. The participant's vital signs were measured after he had rested for at least 5 min in a seated position.

11) Clinical laboratory tests:

- Haematological tests: haematocrit, haemoglobin, MCH, MCHC, MCV, platelet count, RBC count, WBC count, and WBC differential (% and absolute; segmented neutrophils, lymphocytes, monocytes, eosinophils, and basophils).
- Blood chemistry tests: albumin, ALP, ALT, AST, BUN, calcium, chloride, cholesterol creatinine, GGT, glucose, LDH, phosphorus, potassium, sodium, total bilirubin, total CO_2 (bicarbonate), total protein, triglycerides, uric acid, insulin, HbA1c, eGFR, VLDL, and HOMA-IR.
- Urine tests: colour and appearance, pH, specific gravity, bilirubin, glucose, ketone, WBC, nitrite, occult blood, protein, microscopy (RBC and WBC included).

12) Drug abuse tests: Barbiturates, cocaine, opiates, and cannabinoids testing were performed.

13) Serologic tests: HBsAg, HCV Ab, HIV Ag/Ab, and VDRL testing were performed.

14) Pregnancy test: For women of childbearing potential, serum pregnancy tests will be conducted during screening and urine pregnancy tests from the third to the sixth visits.

15) Reproductive hormone tests: Testosterone (total and free), LH, FSH, and SHBG tests were performed.

16) Sampling for genetic research: Participants can participate in this study without participating in the exploratory genetic research part. Participants in exploratory genetic research must sign a consent form for the main study and a separate consent form for experimental genetic research.

17) Pharmacokinetic sampling: Conducted on day 1 pre-dose, day 28 pre-dose, day 56 pre-dose, and day 84 pre-dose.

18) Pharmacodynamic sampling:

- Blood sugar-related: fasting plasma glucose, insulin, C-peptide, HbA1c, and HOMA-IR.
- Endocrine-related: GLP-1 (active/total), GIP, adiponectin, leptin, IL 6, hsCRP, TNF-α, and IL-1β.
- Lipid-related: total cholesterol, LDL-C, HDL-C, VLDL, triglycerides, PON1 (protein quantity, enzyme activity). However, blood sampling for PON1 (protein quantity and enzyme activity) evaluation was performed only on days 1 and 12.

19) Body composition measurement: Skeletal muscle mass (kg), body fat percentage (%), segmental (right arm, left arm, trunk, right leg, and left leg) fat mass (kg), basal metabolic rate (kcal), abdominal fat ratio, and visceral fat level.
